# The Antioxidant and Anti-Inflammatory Properties of Wild Bilberry Fruit Extracts Embedded in Mesoporous Silica-Type Supports: A Stability Study

**DOI:** 10.3390/antiox13020250

**Published:** 2024-02-19

**Authors:** Ana-Maria Brezoiu, Mihaela Deaconu, Raul-Augustin Mitran, Nada K. Sedky, Frédéric Schiets, Pedro Marote, Iulia-Stefania Voicu, Cristian Matei, Laila Ziko, Daniela Berger

**Affiliations:** 1 Faculty of Chemical Engineering and Biotechnologies, National University of Science and Technology POLITEHNICA Bucharest, 1-7 Gheorghe Polizu Street, 011061 Bucharest, Romania; ana_maria.brezoiu@upb.ro (A.-M.B.); mihaela.deaconu@upb.ro (M.D.); iulia.voicu@stud.upb.ro (I.-S.V.); cristian.matei@upb.ro (C.M.); 2 “Ilie Murgulescu” Institute of Physical Chemistry, Romanian Academy, 202 Splaiul Independentei, 060021 Bucharest, Romania; raul.mitran@gmail.com; 3 Department of Biochemistry, School of Life and Medical Sciences, University of Hertfordshire, Hosted by Global Academic Foundation, R5 New Garden City, New Administrative Capital, Cairo 11835, Egypt; nadasedky22@gmail.com (N.K.S.); l.adel@gaf.edu.eg (L.Z.); 4 UMR 5280 CNRS, University Claude Bernard Lyon 1 ISA, 5 Rue de la Doua, 69100 Villeurbanne, France; frederic.schiets@isa-lyon.fr (F.S.); pedro.marote@univ-lyon1.fr (P.M.)

**Keywords:** wild bilberry extract, polyphenolic extract-loaded mesoporous silica, polyphenol stability, anti-inflammatory activity, COX-2 selectivity

## Abstract

Polyphenolic extracts from wild bilberries (*Vaccinium myrtillus* L.) have shown antioxidant and anti-inflammatory effects, but they are prone to degradation when exposed to environmental factors, limiting their use in biomedical applications. To overcome this issue, this study proposed the embedding of wild bilberry fruit ethanolic extracts in pristine mesoporous silica functionalized with organic groups (mercaptopropyl and propionic acid), as well as coated with fucoidan, a biopolymer. Herein, we report a stability study of free and incorporated extracts in mesoporous silica-type supports in high-humidity atmospheres at 40 °C up to 28 days, using HPLC analysis, thermal analysis, and radical scavenging activity determination. Better chemical and thermal stability over time was observed when the extracts were incorporated in mesoporous silica-type supports. After 12 months of storage, higher values of antioxidant activity were determined for the extract embedded in the supports, silica modified with mercaptopropyl groups (MCM-SH), and fucoidan-coated silica (MCM-SH-Fuc) than that of the free extract due to a synergistic activity between the support and extract. All encapsulated extracts demonstrated remarkable effects in reducing NO production in LPS-stimulated RAW 264.7 cells. The treatment with extract embedded in MCM-SH-Fuc in a dose of 10 μg/mL surpassed the effect of free extract in the same concentration. For the extract encapsulated in an MCM-SH support, a lower IC_50_ value (0.69 μg/mL) towards COX-2 was obtained, comparable with that of Indomethacin (0.6 μg/mL). Also, this sample showed a higher selectivity index (2.71) for COX-2 than the reference anti-inflammatory drug (0.98). The developed formulations with antioxidant and anti-inflammatory properties could be further used in nutraceuticals.

## 1. Introduction

Bilberries (*Vaccinium myrtillus* L.) belong to the *Ericaceae* family, which contains more than two hundred species [1]. Wild blueberries are fruits that are usually used fresh, dried, or processed (jams, juice, dietary supplements). They have been utilized to treat mucosal tissue inflammation and skin ulcers and to improve the visual function of the eyes [2]. Bilberries have various health-promoting effects. Different research studies evidenced that fruits, leaves, and/or extracts exhibit beneficial effects such as antioxidant [3,4], anti-obesity [5], anticarcinogenic [6], cardioprotective [7], anti-inflammatory, antimicrobial [3,8], hypoglycemic [9], and vision-improving effects [10]. Several clinical trials of metabolic disorders reported that bilberry consumption as fruits (frozen, processed, or fresh) or juices resulted in a remarkable decrease in inflammatory markers [11,12]. For instance, bilberry juice was noted to reduce the levels of some inflammatory cytokines, including C-reactive protein (CRP) and IL-6 in plasma, hence modulating the inflammatory response [13]. Preclinical examination of mice liver following exposure to the anthocyanins-rich extract from bilberry showed a marked decline in the gene expression of nitric oxide synthase (iNOS), TNF-α, IL-1β, and IL-6 inflammatory markers, along with a subsequent reduction in the levels of iNOS, TNF-α, and NF-κB, which indicated potent protective effects against inflammation of bilberry extracts [14]. Pro-inflammatory mediators and cytokines such as nitric oxide (NO), IL-1β, prostaglandin E2 (PGE2), IL-6, and TNF-α are typically involved in the transformation of the inflammatory response from acute (short duration, characterized by fluid exudation and neutrophils emigration) to chronic (long duration, associated with fibrosis, tissue necrosis, lymphocytes, and macrophages) leading to incurable diseases. Therefore, to design formulations with anti-inflammatory properties, these cytokines and pro-inflammatory factors should be targeted [11].

The main compounds of bilberries that are responsible for the beneficial effects vary depending on the parts of the plant (leaves, fruits) used for the extract preparation. Fruit extracts contain large amounts of anthocyanins (aprox. 90% wt. polyphenolic compounds) derived mainly from cyanidin and delphinidin in glycosylated forms of glucosides, galactosides, or arabinosides. Besides these, flavonoids (quercetin, myricetin), flavanols (catechin, epicatechin, procyanidins dimers, or trimers), stilbenoids (resveratrol), organic acids (shikimic, quinic, citric, and malic acids), phenolic acids, and hydroxycinnamic acid derivatives (*p*-coumaric, caffeic, ferulic acids) could also be found [15]. The main phytocompounds in leaf extracts are catechins (catechin, epicatechin, gallocatechin, epigallocatechins), cinchonains I and II, phenolic acids (mainly *p*-coumaric, chlorogenic, and caffeic acids), proanthocyanidins, and flavonols (quercetin, rutin, kaempferol, and their derivatives) [3,16].

The thermal stability of individual polyphenolic compounds or bilberry extracts was tested regarding the preservation of beneficial properties over time. The low anthocyanins stability at elevated temperatures was already proven, and their degradation follows a first-order kinetics (k = 2.999 × 10^−3^ days at room temperature compared to k = 0.263 days at 60 °C). However, the half-life of bilberry extract at room temperature is about 231 days, and the addition of antioxidants (ascorbic acid or butylated hydroxyanisole) does not improve their stability over time [17]. Another study reported the long-term stability of anthocyanins from bilberry methanolic extract. The results showed that after 4 months of storage, a 7% reduction in the initial content at 4 °C was noticed, compared to 83% degradation of the initial content that occurred when stored at 25 °C in dark conditions [18]. An increased anthocyanin stability in simulated gastric fluid compared to simulated intestinal fluids (about 60% loss of anthocyanin) was also reported [19].

Due to the low stability of anthocyanins, it is imperative that the extracts are encapsulated to preserve their beneficial properties over time. Several reports have been published on the entrapment of bilberry extract in various matrices. For instance, arabic gum and maltodextrin were employed to encapsulate bilberry extract, and an improved stability up to 100 °C was proven, the obtained formulations being recommended as a food additive [20]. Another study reported the encapsulation of bilberry extract in zein protein through electrospray drying [21]. Our group reported the embedding of polyphenolic extracts from various vegetal sources into mesoporous materials, leading to a better stability of the extracts over time [22,23,24,25]. Moreover, mesoporous silica-type supports would protect the extracts and could target certain cells, depending on the functionalization of the silica surface.

Herein, we report the incorporation of wild bilberry fruit extracts into mesoporous silica pristine, functionalized with organic groups, and coated with a natural polymer, fucoidan. We chose pristine mesoporous silica and functionalized with organic groups as supports for the bilberry extracts due to their advantages over other types of inorganic nanoparticles (ZnO, Fe_3_O_4_, carbon nanotubes, metallic nanoparticles), such as high porosity, which means high loading capacity with organic molecules, superior biocompatibility, and biodegradability [26], and the possibility to bind on silica surface functional groups that modulate cargo molecule–support interactions. Compared to organic supports like liposomes, polymers, maltodextrin, and arabic gum, silica has superior chemical and photo-stability, which are important characteristics for topical applications. Silica is approved by the Food and Drug Administration (FDA) for cosmetics, as a food additive, or in food packaging [27,28].

Developed formulations based on bilberry fruit extract showed preservation or better radical scavenging activity than the free extract, as well as anti-inflammatory properties with good selectivity for COX-2, highlighting good anti-inflammatory potential without having the side effects associated with COX-1 enzyme [29]. Furthermore, we report a stability study of free and incorporated extracts in mesoporous silica-type supports in a high-humidity atmosphere at 40 °C for up to 28 days, using HPLC-PDA analysis, thermal analysis, and radical scavenging activity determination.

## 2. Materials and Methods

### 2.1. Materials

All chemicals (reagent grade) used in this study were purchased from Sigma-Aldrich (Merck Group, Darmstadt, Germany) or TCI Chemicals (Tokyo, Japan) and were used as received. For high-performance liquid chromatography (HPLC), solvents and twenty-three standard compounds (HPLC grade) were used.

Freeze-dried wild *Vaccinium myrtillus* fruits from the Cindrel Mountains (1700 m altitude) were used as vegetal material.

### 2.2. Methods

#### 2.2.1. Extract Preparation and Characterization

Wild bilberry fruit extracts were prepared in ethanol acidified with citric acid (10^−2^ M citric acid) at 80 °C using conventional method (BL extract) or solvothermal treatment (BL(ST) extract). Conventional extraction was performed in four extraction stages of 1 h each, with solvent replacement (total of 1/24 g/mL fruit/solvent ratio) and solvothermal extraction under 5 atm argon pressure, in one stage of three hours (1/18 g/mL fruit/solvent ratio), using a reactor with external temperature controller (Roth, KarlsRuhe WRX 2000, Frederikssund, Denmark). The solid was filtered off, and the supernatant was evaporated under a vacuum until it achieved a constant dried mass. For further analyses, the extract was dissolved in the extraction solvent.

The process yield was computed using Equation (1):

(1) $$Yield\;(\%)=\frac{m_{de}}{m_{df}}\cdot 100$$

where *m_de_* is the mass of dried extract (g), and *m_df_* is the mass of dried fruits (g).

Spectrophotometric analyses (Shimadzu UV-1800, Kyoto, Japan) comprising total polyphenols, flavonoids, anthocyanins, and radical scavenger activity through DPPH and ABTS assays were carried out as previously reported, and the chemical profile of the extracts was determined using reverse-phase high-performance liquid chromatograph Shimadzu Nexera 2 with a multi-wavelength UV detector (SPD-M30A) in 250–600 nm wavelength range [22,23,30]. The identification of extract compounds was carried out based on retention time and spectrum similarity.

The anthocyanins analysis was carried out by liquid chromatography–mass spectrometry (LCMS, Agilent Technologies 1200 with G1379B degasser, G1312B binary pump, and G6125B mass detector) using a Kinetex 2.6 mm C18 column (Phenomenex, Danaher, Washington, DC, USA) with 100 Å pore size, 150 × 3 mm, using an elution program (0.4 mL/min) at 35 °C and an injection volume of 1 µL. The mobile phases were (A) ACN/HCOOH = 100/0.1% (*v*/*v*) and (B) H_2_O/HCOOH = 100/10% (*v*/*v*). The elution gradient was as follows: 6 min isocratic elution using mobile phase B 92%, then 14 min decreased linearly to 65% mobile phase B, 10 min linear gradient 0% mobile phase B; 2 min isocratic elution 0% mobile phase B; and 4 min linear increase to the initial conditions. The mass data were recorded at 520 nm wavelength. The mass selective detector (MSD) was set for full scan from 100 to 600 *m*/*z* in positive API-ES polarity mode with 70 eV fragmentation energy and a 3 kV capillary voltage. Nitrogen was used as nebulizer at 35 psig and 350 °C. Desolvation gas (nitrogen) with a temperature of 350 °C was delivered at a constant flow (12 L/min).

The stability of the bilberry fruit extracts, free or embedded in mesoporous silica, was evaluated in relative humidity (RHu) of 75%. Open vials with samples were placed in a desiccator with a Petri dish containing saturated solution of NaCl and exposed to 40 °C for up to 28 days [3]. The time set intervals and temperature were chosen after consulting the recommendation of World Health Organization regarding stability studies. The samples were subsequently characterized by thermogravimetric analysis coupled with differential thermal analysis (GA/SDTA851e, Mettler Toledo, Greifensee, Switzerland), HPLC-PDA, and spectrometric determination of radical scavenger activity.

#### 2.2.2. Obtaining Mesoporous Silica-Type Matrices and Extract-Loaded Materials

MCM-41 mesoporous silica nanoparticles were synthesized by a previously described sol-gel method, using tetraethyl orthosilicate (TEOS) as precursor, triethanolamine (TEA) as catalyst, cetyltrimethylammonium chloride (CTAC) as template agent, and poly(ethylene glycol) methyl ether (PEG) to end the condensation reactions of silicate intermediates [31]. MCM-41 support was calcined at 550 °C, 5 h.

Mercaptopropyl functionalized mesoporous silica (MCM-SH) was obtained by co-condensation approach from TEOS and (3-mercaptopropyl) triethoxisylane (MPTES). TEOS and MPTES were mixed and added to TEA and then kept at 90 °C for 20 min without stirring. Next, cetyltrimethylammonium bromide (CTAB) previously dissolved in water was poured into the mixture containing TEOS and organosilane, followed by the addition of PEG aqueous solution (50 mg/mL) under stirring. The reaction was kept at reflux for 18 h. The TEOS:MPTES:TEA:CTAB:PEG:H_2_O molar ratio used in the synthesis was 0.9:0.1:0.2:10.4:0.05:130. Functionalized silica nanoparticle suspension was poured in ethanol and then separated by centrifugation. CTAB was removed through ultrasound-assisted extraction in ethanolic solution of NH_4_Cl (7 g/L) in two steps of 1 h, at 60 °C, with intermediate nanoparticle separation.

Mesoporous silica functionalized with propionic acid moieties (labeled MCM-COOH) was obtained by post-synthesis approach using pure mesoporous silica and 3-(triethoxysilyl)propionitrile in 1/0.25 molar ratio as described elsewhere [31]. To convert propionitrile groups into propionic acid moieties, a hydrolysis reaction was carried out in aqueous sulfuric acid solution (50% wt, 20 mg/mL) at reflux for 4 h.

To obtain MCM-SH-Fuc support, MCM-SH nanoparticles were coated with fucoidan. Thus, 100 mg of MCM-SH was added to 10 mL of fucoidan solution (5 mg/mL, aq.) under magnetic stirring at 20 °C for 40 min. Then, 40 mL of ethanol was added dropwise at 5 °C, under magnetic stirring, and finally, the solid was separated by filtration.

Extract-loaded samples were obtained through an impregnation method. The mesoporous silica-type support, outgassed at 3 mbar for 12 h, was impregnated with the ethanolic extract (15 mg/mL). After homogenization of the mixture, the solvent was removed under vacuum at 3 mbar. The samples were denoted BL@support and BL(ST)@support.

#### 2.2.3. Characterization of Carriers and Extract-Loaded Materials

Mesoporous carriers were analyzed by nitrogen adsorption–desorption isotherms using Micromeritics TriStar II Plus gas sorption analyzer (Micromeritics Instrument Corporation, Norcross, GA, USA).

The structural features of mesoporous supports were assessed by small-angle X-ray diffraction (XRD) on a Rigaku MiniFlex II (Rigaku Corporation, Tokyo, Japan) in 1.2–6.0 2*θ* range.

FTIR spectroscopy was used to evidence the silica surface functionalization. FTIR spectra (Bruker Tensor 27, Bruker Corporation Optik GmbH, Bremen, Germany) of the samples, as KBr pellets, were recorded in the range of 400–4000 cm^−1^.

The organic group content on the silica surface and the extract amount from extract-loaded samples were assessed by thermogravimetric analysis (TGA, Netzsch STA 2500 Regulus, Selb, Germany) in 30–800 °C domain with 10 °C/min heating rate under synthetic air.

The thermal behavior of wild bilberry extracts and extract-loaded materials was assessed using a differential scanning calorimeter from Mettler Toledo (Greifensee, Switzerland) DSC 823e equipped with a digital camera and operated under constant nitrogen flow (80 mL/min) and a heating rate of 10 °C/min between −20 °C and 150 °C.

The morphology of supports was evaluated by scanning electron microscopy (SEM, Tescan Vega 3 LMH microscope, Brno, Czech Republic) and transmission electron microscopy (TEM, TECNAI F30 G^2^ S-TWIN, FEI, Hillsboro, OR, USA).

#### 2.2.4. Anti-Inflammatory Assay in Mouse Macrophage Cells

Mouse macrophage cell line (RAW264.7) was acquired from Nawah Scientific Inc. (Mokatam, Cairo, Egypt). The cells were grown in DMEM media supplied with streptomycin (100 mg/mL), penicillin (100 units/mL), and 10% of heat-inactivated fetal bovine serum in humidified atmosphere. The cells were placed in a 5% CO_2_ incubator, and temperature was adjusted to 37 °C. After preincubation for 1 h, 10 and 100 µg/mL of each sample were added. Cell viability of RAW 264.7 cells was assessed after 72 h exposure to the test compounds by SRB assay. Briefly, a 96-well plate was used to culture the cells. A 100 μL cell suspension (5 × 10^3^ cells) was placed in each well. Then, 100 μL media of test compounds at the required concentrations were added to the wells. On the third day, a fixation step was performed where the media was replaced with 150 μL of 10% TCA and preserved at 4 °C for 1 h. Directly afterward, five consecutive rinsing steps with distilled water were applied, and 70 μL SRB solution (0.4% *w*/*v*) was added to every single well. The plates were kept in dark conditions at 25 °C for 10 min. Plates were then rinsed with 1% acetic acid and air-dried overnight. Thereafter, in each well, an aliquot of 150 μL of TRIS (10 mM) was added. A BMGLABTECH^®^- FLUOstar Omega (Ortenberg, Germany) microplate reader was used to measure the absorbance at 540 nm.

The levels of NO were analyzed from the cell culture supernatants as prescribed elsewhere [32]. In brief, an estimate of 1 × 10^5^ RAW 264.7 cells were cultured and incubated in each well of a 96-well plate for 24 h. The following day, two different concentrations of the samples (10 and 100 μg/mL) were added to the cells an hour prior to the addition of LPS. Untreated cells were employed as the control group. LPS was then added to produce inflammation in the cells. Cells were then incubated with the test samples and LPS for 24 h. Lastly, cell culture supernatants were mixed with Griess reagent in dark conditions (10 min) at room temperature, and nitrite absorbance was read at 540 nm.

#### 2.2.5. COX Enzyme Inhibition Assay (In Vitro Screening)

The tested samples and Indomethacin, used as reference, were examined for their ability to inhibit the enzymes COX-1 and COX-2 using COX Inhibitor Screening Assay kit (catalog # 560131, Cayman ACE™, EIA kits, Michigan, USA). Serial dilutions ranging from 0.01 μg/mL to 100 μg/mL of the tested samples were used to obtain the dose–response inhibition curve and compute the concentration of the test compound, which causes 50% inhibition (IC_50_, μM).

## 3. Results and Discussion

### 3.1. Polyphenolic Extracts Characterization

Both conventional and solvothermal extraction methods are efficient considering the process yield (Table 1), which was higher than 70%. A slightly higher value was achieved for the solvothermal method; probably, a higher pressure facilitates the extraction process. The extraction yields from bilberry pomace reported by Piechowiak et al. were lower, being in the range of 12.41–39.80%, because of the use of a residue as raw material [33]. Good yields (65.08–77.78%) were obtained for bilberry extracts prepared by microwave-assisted extraction using 100–300 W power and a solid/liquid ratio of 1/5–1/50, which are consistent with our values [34].

The total polyphenol content (TPC) was similar for both extracts (45.82–46.46 mg gallic acid equivalents (GAE)/g DW). This content is larger than that of Piechowiak et al. for ethanolic ultrasound-assisted extracts obtained from bilberry pomace (5.3–41.8 mg GAE/g DW) [33]. Secco et al. reported the influence of pressurized liquids on the extraction of polyphenols from bilberry pomace, the best results being obtained for 20% propylene glycol aqueous solution. This extract had a TPC of 41.16 ± 0.23 mg GAE/g DW, lower than the values reported in this study [35]. The geographic origin of fruits, extraction temperature, and solvent greatly influence the composition of extracts. For instance, Huang et al. reported a TPC of 9.44 ± 0.22 mg GAE/g DW for 80% methanolic extracts, a lower value than presented herein [36].

The total flavonoid content (TFC) of our extracts (14.45–18.79 mg RHE/g DW) is lower than in the case of a methanolic extract obtained at room temperature (36.08 ± 0.56 mg RE/g DW) [36].

A higher total anthocyanin content was yielded in our extracts (28.86–32.25 mg CGE/g DW), probably due to an acidic solvent medium, compared to previously reported extracts prepared by microwave-assisted extraction (5.14–8.80 mg CGE/g DW) [37]. However, Bunea et al. obtained similar results for acidified methanol extracts from wild bilberries from the Romanian mountains (25.02–30.02 mg CGE/g DW) [37].

The radical scavenging activity of bilberry extracts is in the range of 115.49–119.89 mg TE/g DW for DPPH assay (461.43–479.01 μmol TE/g DW) and 60.44–72.00 mg TE/g DW for ABTS assay (241.46–287.69 μmol TE/g DW), respectively. Jurca et al. reported higher values for the ABTS method for 70% ethanolic extracts from bilberries grown in Romania, Bihor County (275.55–327.63 μmol TE/g DW) [38]. However, our values are higher than those reported by Rodrigues (18.50 ± 4.04 μmol TE/g DW, ABTS assay) for methanolic extracts prepared through ultrasound-assisted extraction [39].

Up to eight compounds from the twenty-three available standard substances were quantified in the extracts by HPLC-PDA (Figure S1, Supplementary Materials). The identified compounds from the polyphenolic acids group in 100 g of dried fruits (DW) are gallic acid (3.419–3.425 mg), chlorogenic acid (27.269–28.154 mg), and protocatechuic acid found only in the BL(ST) extract (3.818 ± 0.027 mg) (Table 2). From the anthocyanidins class, delphinidin was quantified (153.645–165.840 mg) in both extracts, being in a larger amount in the solvothermal extract than in BL, and cyanidin in a small amount and only in BL (1.588 ± 0.015 mg). In the extracts, two flavonoids were identified: rutin hydrate (95.113–101.379 mg) and myricetin (6.484–7.015 mg), both found in greater quantity in the solvothermal extract. Also, low levels of *trans*-resveratrol were identified in both extracts in the range of 1.036–1.062 mg. Mustafa et al. reported for bilberry extracts the following compounds (expressed in the same units as our values): gallic acid (0.78 ± 0.08 mg), chlorogenic acid (34.40 ± 0.02 mg), rutin (3.45 ± 0.12 mg), myricetin (0.3 ± 0.02 mg), with all amounts being lower than that of our extracts [40]. The resveratrol content reported by Seyhan et al. was between 0.34 and 1.00 mg/100 g DW for methanolic extracts from bilberries of different species [41].

Anthocyanin glycoside identification was carried out by LCMS based on a molecular mass determination, their retention times, and literature data [37]. Eight anthocyanin glycosides were identified in BL extracts, being rich in delphinidin-3-*O*-glycosides and cyanidin-3-*O*-glycosides (as galactoside, glucoside, and arabinoside). Among anthocyanin glycosides, petunidin-3-*O*-glucoside and malvidin-3-*O*-galactoside were also found. The identified anthocyanin glycosides, their retention times, and molecular weights can be found in the Supplementary Materials (Table S1).

### 3.2. Stability Study of Free Extracts

Natural extracts are prone to degradation when exposed to environmental factors. Therefore, it is crucial to evaluate their stability over time in order to assess how long they preserve their beneficial properties [42]. A 28-day study was performed to determine the extract’s stability. Samples were taken at different time intervals and then tested for volatile compounds, organic components, and their residues by thermogravimetric analysis, as well as their chemical profile through HPLC-PDA analysis.

An increase in volatile components was noticed during the 28-day incubation in a high-humidity atmosphere (Table 3). The conventional extract had lower amounts of volatile components, probably because it was prepared under reflux, where some of the volatile compounds were lost to the atmosphere, in contrast to the BL(ST) extract, which was prepared under argon pressure in a closed reactor.

Similar water uptake was noticed for both extracts, up to 22–24% during the 28-day incubation. A much lower water absorption (up to 15%) was observed for BL@MCM-41, the mesoporous support acting as a protective barrier against environmental factors. Therefore, the extract is better protected when confined in silica mesopores.

**Figure 1:**
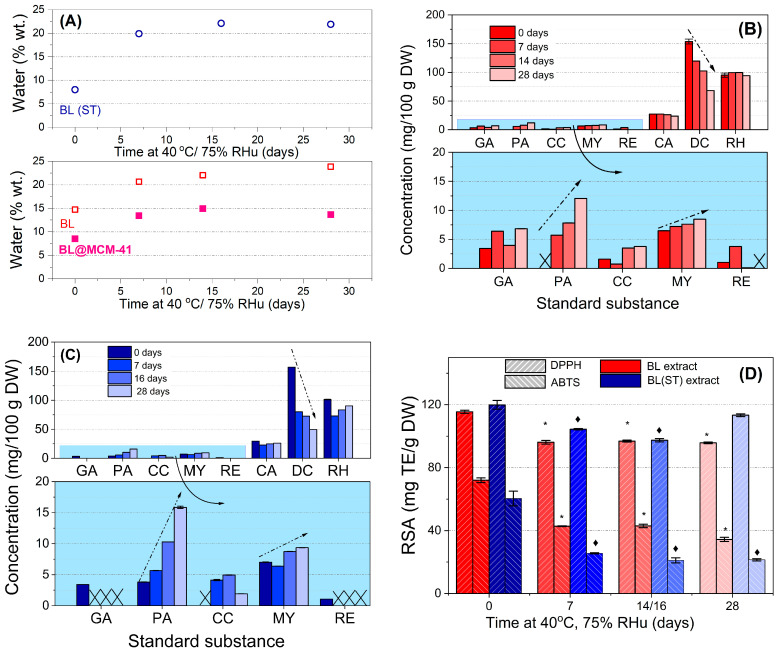
Results of the accelerated degradation study of the bilberry extracts: (**A**) water amount from TG-DTA; (**B**,**C**) results quantified from HPLC–PDA data for BL extract and BL(ST) sample, respectively (GA—gallic acid; PA—protocatechuic acid; CC—cyanidin; MY—myricetin; RE—resveratrol; CA—chlorogenic acid; DC—delphinidin; RH—rutin hydrate); (**D**) radical scavenging activity (RSA) expressed as Trolox before and after 7, 14, and 28 days of treatment. The symbol * or ♦ shows a significant difference (with a value of *p* < 0.05) to the corresponding untreated free BL extract or BL(ST) sample, respectively. The arrows show the concentration variation.

By HPLC-PDA analysis of extracts before and after 7–28 days of accelerated degradation treatment at 40 °C and 75% RHu (Figure 1B,C), up to eight compounds were identified in both extracts, larger quantities of delphinidin chloride (68.183–153.645 mg for BL and 49.591–157.146 mg for BL(ST) extract), rutin hydrate (94.185–99.171 mg for BL and 83.522–101.379 mg for BL(ST) extract), and chlorogenic acid (23.820–27.869 mg for BL and 22.658–29.363 mg for BL(ST) extract) were found. All values were presented per 100 g of dried fruits. During the stability study, the decrease in the content of delphinidin was associated with an increase in the amount of cyanidin due to the possible hydrolysis and subsequent degradation of this anthocyanidin. Enhanced contents of protocatechuic acid and myricetin and reduced amounts of resveratrol, chlorogenic, gallic, and rutin hydrate were also determined [43].

The variation of radical scavenging activity of extracts prior to and after different incubation periods during the accelerated degradation study can be seen in Figure 1D. The RSA values are significantly different from those of the untreated extract. An important reduction in the RSA values of BL and BL(ST) extracts (5.5–18.73%—DPPH method or 40.40–65.13%—ABTS assay) was observed due to the response of the extract to environmental factors [44]. Hence, there is an inherent need for encapsulation to maintain the beneficial properties of fruit extracts.

### 3.3. Characterization of Mesoporous Silica-Type Supports

BL polyphenolic extract was loaded in pure and functionalized MCM-41 supports.

**Figure 2:**
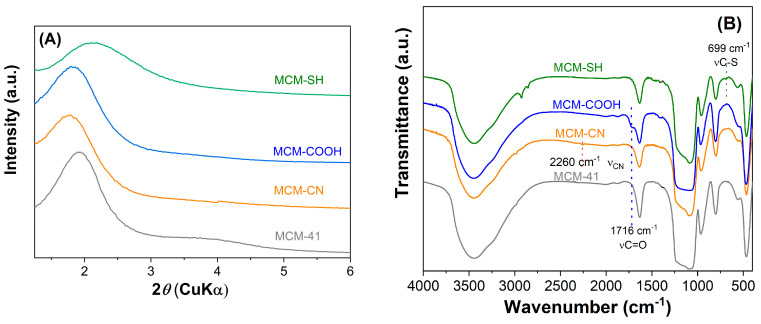
Small−angle XRD patterns (**A**) and FTIR spectra (**B**) for pristine and functionalized mesoporous materials.

The mesoporous silica-type materials were analyzed by various techniques. Small-angle X-ray diffraction patterns (Figure 2A) demonstrated that both pristine and functionalized supports had a hexagonal pore array, all supports showing at least the diffraction peak associated with the (100) Bragg reflection. After the functionalization reaction of the silica surface with organic groups, a shift towards higher *d_100_*-spacing values was noticed, unlike the case of MCM-SH support, which was prepared by the co-condensation method, whose pore framework is less ordered because of the high content of functional groups.

The FTIR spectra evidenced the modification of silica surfaces with organic moieties. All MCM-41 silica supports (Figure 2B) exhibit specific bands of silica matrix at 1084 and 804 cm^−1^, silanol vibration at 968 cm^−1^, Si–O bond deformation at 468 cm^−1^, and also the band of physisorbed water at 1640 cm^−1^. The spectra of functionalized silica materials (Figure 2B) contain aliphatic methylene stretching vibrations in the 2950–2800 cm^−1^ region.

In the FTIR spectrum of MCM-COOH synthesized through hydrolysis reaction from MCM-CN material, one can observe a sharp band (stretching vibration of C=O bond) at 1716 cm^−1^ and no vibration at 2260 cm^−1^ (C–N stretching), demonstrating a complete conversion of -CN groups in -COOH moieties. In the FTIR spectrum of MCM-SH, the stretching vibration from 699 cm^−1^ was associated with C-S bonds.

**Figure 3:**
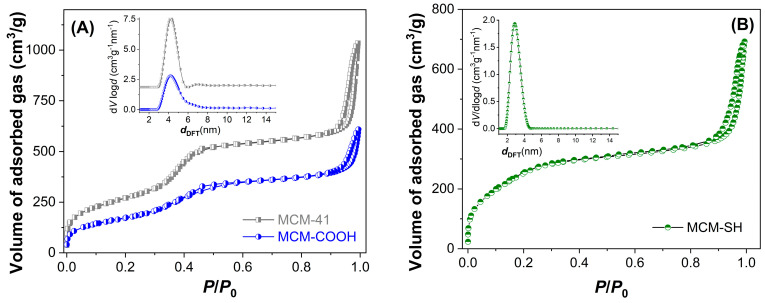
N_2_ adsorption−desorption isotherms and the corresponding pore size distribution curves calculated with DFT (inset) for MCM−41 and MCM−COOH (**A**) and MCM−SH (**B**).

An important parameter in developing extract-loaded materials is the supports’ porosity. The textural characteristics of mesoporous supports were determined from isotherms. The pore diameter distribution curves (Figure 3 inset), as well as average pore diameters, were determined using DFT [23]. These parameters are listed in Table 4. After functionalization, the porosity and the average pore diameter decreased compared to pristine silica used for the synthesis of MCM-COOH (Figure 3A). MCM-SH material exhibited a higher porosity, although it had a high content of organic groups (Figure 3B). The functionalized silica samples had a lower specific surface area (628–933 m^2^/g) and pore volume (0.93–1.19 cm^3^/g) values than silica (989 m^2^/g and 1.59 cm^3^/g, respectively), but they had enough porosity to accommodate a high amount of polyphenols from the extract.

The content of organic moieties for silica supports was assessed by TG-DTA based on the mass loss in the temperature range of 120–600 °C, the first endothermic event being attributed to the adsorbed water molecules.

**Figure 4:**
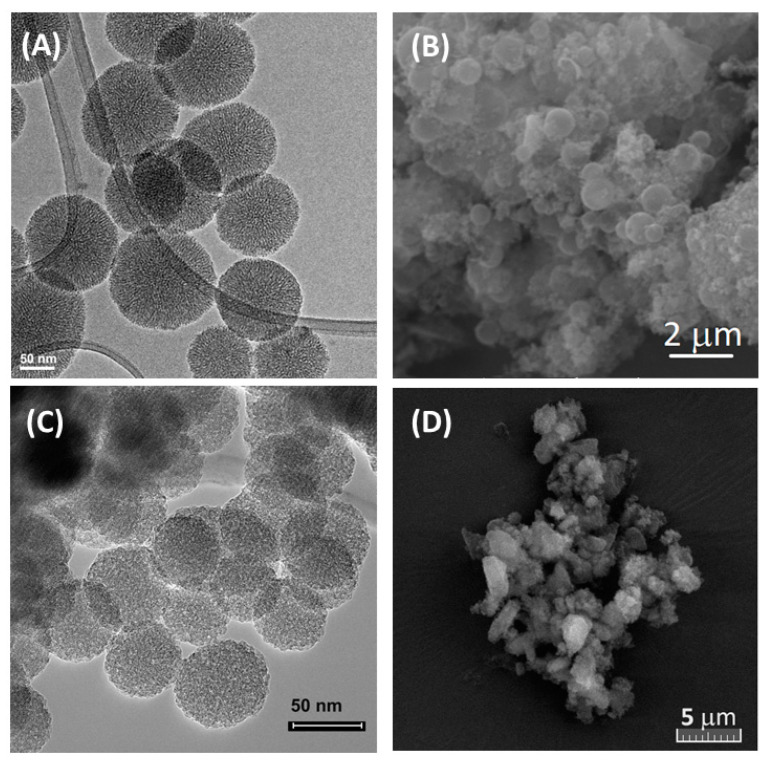
TEM micrographs of MCM-41 (**A**) and MCM-SH (**C**) and SEM images of MCM-COOH (**B**) and MCM-SH-Fuc (**D**).

The MCM-41 carriers were characterized by SEM and TEM investigation. MCM-41 sample presents spherical-shaped particles with a diameter of 90–140 nm (Figure 4A). MCM-COOH obtained from pristine MCM-41 has more agglomerated particles (Figure 4B). MCM-SH synthesized by the co-condensation method has smaller spherical nanoparticles than MCM-41, having a diameter of 40–60 nm (Figure 4C). For MCM-SH-Fuc, SEM investigation revealed the presence of polymer on mercaptopropyl functionalized silica nanoparticles forming agglomerates with irregular shapes (Figure 4D).

### 3.4. Characterization of Embedded Bilberry Extracts

Extract-loaded materials were analyzed by thermogravimetric analyses, differential scanning calorimetry (DSC), and radical scavenger activity. Based on thermogravimetric analyses (Supplementary Materials, Figure S2), the content of polyphenolic compounds was determined considering the contribution of the organic groups grafted on silica surfaces. The extract-loaded materials have a content of phytocompounds in the range of 20.2–41.6% wt., which depends on the carrier’s total pore volume (Table 5).

**Figure 5:**
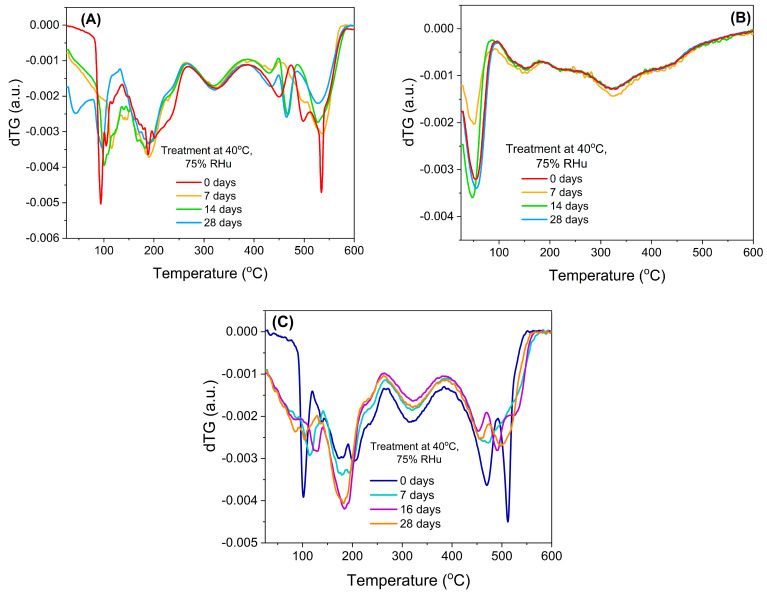
First derivative of thermogravimetric analyses (dTG): (**A**) BL extract, (**B**) BL extract embedded in MCM−41 support, and (**C**) BL(ST) free extract.

The chemical stability of bilberry extracts, when loaded into mesopores of silica-type support, could also be evaluated from the first derivative of the thermogravimetric curves (*d*TG), which revealed that the free extracts (Figure 5A,C) were prone to a faster degradation when exposed to high humidity, while the encapsulated extract (Figure 5B) showed a better chemical stability.

**Figure 6:**
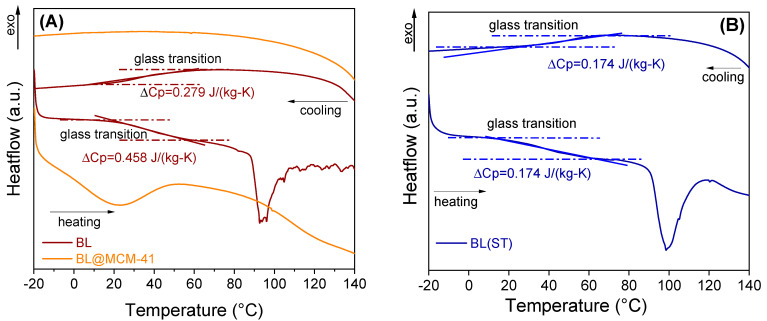
DSC analysis of free BL extract and embedded in MCM−41 support (**A**) and BL(ST) extract (**B**).

Evaluation of the thermal stability was carried out using a differential scanning calorimeter equipped with an optical microscope. For the free extract, a type II glass transition was noted that occurred with a specific heat change, computed at the inflection point, showing the transition from an amorphous state to a supercooled liquid, while the encapsulated extract did not present a glass transition in the tested temperature range (Figure 6). For the encapsulated extract, no important changes were noticed up to 150 °C. The videos can be seen in the Supplementary Materials (Figure S3).

**Figure 7:**
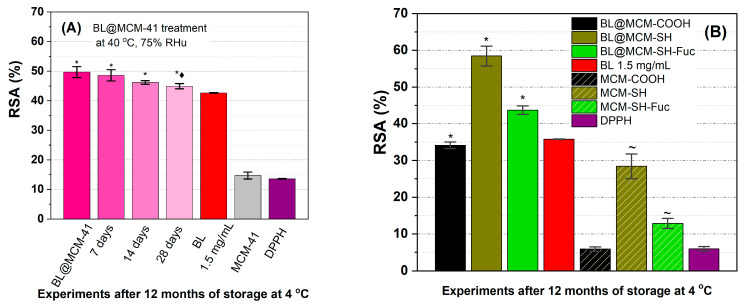
Radical scavenging activity for (**A**) BL alone or embedded in MCM-41 support after accelerated degradation treatment vs. free extract stored at 4 °C and support and (**B**) free BL extract or embedded in functionalized silica supports. Data presented are the average of three runs ± standard deviation (SD). The symbols *, ♦, and ~ refer to significance levels of *p* < 0.05 from the free extract, BL@MCM-41, at the same concentration tested and DPPH, respectively.

The determination of the radical scavenging activity (Figure 7A) showed a better stability of the incorporated extract than that of the free extract. During the accelerated degradation study, the encapsulated extract in MCM-41 support did not differ significantly from the untreated sample during the first 14 days of treatment, with a loss of antioxidant activity of 2.2% (7 days), 7.1% (14 days), and 9, 6% (28 days), all samples having a better radical scavenging activity than the free extract stored at 4 °C. Furthermore, BL@MCM-SH and BL@MCM-SH-Fuc showed enhanced radical scavenging activity due to the synergistic effects between the extract and the support. Compared to BL, BL@MCM-COOH showed a slight decrease in the RSA after 12 months at 4 °C.

### 3.5. Biological Evaluation of Free and Embedded Bilberry Extract

#### 3.5.1. NO Production Inhibitory Effect and Cytotoxicity

RAW 264.7 cells were exposed to the samples at two different concentrations (10 μg/mL and 100 μg/mL) and kept for 1 h and then treated with LPS (1 μg/mL). The supernatant (100 μL) was withdrawn, and NO production was assessed using the Griess reagent. Thereafter, cell viability was determined using SRB assay.

Almost all the tested samples displayed no apparent cytotoxicity at the two investigated concentrations. Only BL and BL@MCM-SH-Fuc treatments induced minimal cytotoxic effects. The application of BL at both 10 and 100 μg/mL resulted in about a 10% reduction in cell viability. BL@MCM-SH-Fuc caused about a 10% and a 15% reduction in cell viability at concentrations of 10 and 100 μg/mL, respectively. Therefore, the safety of the newly proposed formulas is highly assured.

**Figure 8:**
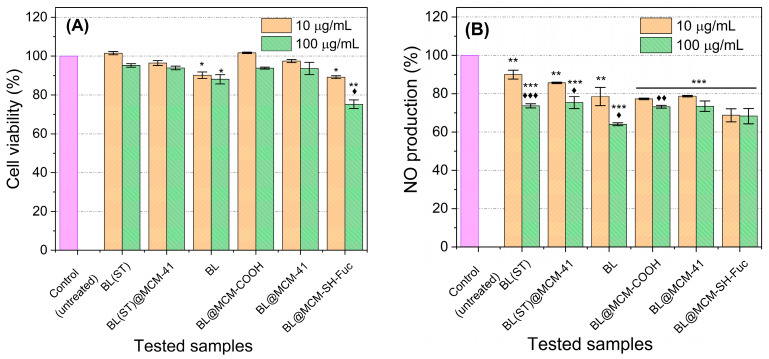
Effects of the test samples on cell viability and NO production. (**A**) RAW 264.7 cell viability of both treated and untreated cells after induction of inflammation by LPS. (**B**) NO production of untreated (exposed to LPS only) and those that were incubated with different test compounds an hour before receiving LPS. Data presented are the mean of three different runs ± standard deviation (SD). The symbols * and ♦ refer to significance of the untreated (control) and the 10 µg/mL concentration of the test compound. Each symbol used once reprezents *p* < 0.05. Each symbol repeated twice (** or ♦♦) and three times (*** or ♦♦♦) times refers to *p* < 0.01 and *p*< 0.001, respectively.

All investigated compounds caused a significant inhibition in NO production for RAW 264.7 cells stimulated with LPS (Figure 8). The most powerful NO inhibition (36% inhibition) was observed upon exposure to BL at a dosage of 100 μg/mL as compared to the control (*p* < 0.001). The application of BL@MCM-SH-Fuc at a dose of 10 μg/mL surpassed all other treatments when applied in the same concentration, resulting in 31% NO inhibition, almost equal in effect to the treatment with 100 μg/mL. BL@MCM-COOH resulted in significant inhibition of NO production—a roughly 23% and 27% reduction in NO production when concentrations of 10 and 100 μg/mL, respectively, were applied. In addition, the material did not show any cytotoxicity. As such, the application of the novel formulations BL@MCM-SH-Fuc and BL@MCM-COOH at low concentrations (10 μg/mL) was successful in inhibiting inflammation and reducing NO production while conserving normal cells.

#### 3.5.2. In Vitro COX inhibition assay

The tested samples, as well as the reference drug Indomethacin, were tested for their ability against COX-1 and COX-2. The IC_50_ value was determined for each of the investigated samples against both COX-1 and COX-2, as shown in Table 6. Our findings showed that BL and BL@MCM-SH formulations were potent inhibitors of COX-2 with IC_50_ values of 0.97 and 0.69 μg/mL, respectively. The computed IC_50_ values of BL and BL@MCM-SH against COX-1 were 3.97 and 1.87 μg/mL, respectively. This, in turn, indicated that the new formulations presented a better selectivity toward COX-2 than for COX-1. The investigated samples demonstrated a selectivity index for COX-2 of approximately 4 (BL) and 3 (BL@MCM-SH) (Table 6). This renders them of good therapeutic potential as anti-inflammatory agents that would be more selective to COX-2 and thus inhibit the inflammation with minimal side effects and without affecting the gastric mucosa [29]. These results could be explained by the high amount of anthocyanin present in the extracts, which already have been proven to exert a reduction of the expression levels of nitric oxide synthase, COX-2, IL-1β, as well as IL-6, therefore reducing inflammation [45]. Other authors have reported that raspberry extracts and fractions also have anti-inflammatory effects, with these being tested on leukemia cells (HL-60, J45.01) and their results being similar to ibuprofen [46].

Due to the antioxidant and anti-inflammatory properties of proposed formulations, bilberry extract could be further applied in cosmetics or nutraceuticals [2,10,15]. The use of mesoporous silica as carriers resulted in good chemical and thermal stability of the natural compounds from the extract that are otherwise prone to degradation under environmental conditions.

## 4. Conclusions

The extracts prepared from bilberry in acidified ethanol had high antioxidant activity because they were rich in anthocyanins. The extracts were unstable, and an important loss of beneficial compounds was noticed through an accelerated degradation study. To overcome this issue, the extracts were incorporated in mesoporous silica-type supports, and an improved stability was achieved over time. This was demonstrated through the radical scavenger activity assessment. Also, the proposed formulations exhibited anti-inflammatory properties with a good selectivity towards COX-2, therefore achieving anti-inflammatory activity with diminished associated side effects while having little to no toxicity.

The synthetic antioxidants or anti-inflammatory agents, usually used in cosmetics or nutraceuticals, could be replaced by extracts incorporated in silica-type supports, which demonstrated good chemical and thermal stability over time.

## Figures and Tables

**Table 1:** Extract yield and spectrophotometric evaluation of bilberry polyphenolic extracts.

Sample	Yield (%)	TPC (mg GAE/g)	TFC (mg RHE/g)	TAC (mg CGE/g)	RSA_DPPH_ (mg TE/g)	RSA_ABTS_ (mg TE/g)
BL	70.5	45.82 ± 0.99	14.45 ± 0.05	28.86 ± 0.82	115.49 ± 0.97	72.00 ± 1.50
BL(ST)	73.8	46.46 ± 0.73	18.79 ± 0.26	32.25 ± 0.32	119.89 ± 2.80	60.44 ± 1.67

BL and BL(ST)—conventional bilberry extract and solvothermal bilberry extract, respectively; TPC—total polyphenol content as gallic acid (GAE); total flavonoid content as rutin hydrate (RHE); TAC—total anthocyanin content in cyanidin-3-glicoside (CGE); RSA—radical scavenger activity in Trolox equivalents (TE). All values are presented in units of grams of fruit dried weight.

**Table 2:** Chemical composition of bilberry extracts determined by HPLC-PDA.

Concentration (mg/100 g DW)	BL	BL(ST)
gallic acid (1)	3.419 ± 0.010	3.425 ± 0.001
protocatechuic acid (2)	nd	3.818 ± 0.027
chlorogenic acid (3)	27.269 ± 0.004	28.154 ± 0.074
delphinidin (4)	153.645 ± 4.298	157.146 ± 0.073
cyanidin (5)	1.588 ± 0.015	nd
rutin hydrate (6)	95.113 ± 3.641	101.379 ± 0.901
myricetin (7)	6.484 ± 0.051	7.015 ± 0.048
*trans*-resveratrol (8)	1.036 ± 0.002	1.062 ± 0.003

nd—not detected.

**Table 3:** Components with high volatility, organics content, and residue mass of bilberry extracts after different incubation times.

Sample	Exposure Time (days)	Components with High Volatility	Organics Content	Residue
		(%wt. vs. DW)		
BL	0	40.3	57.0	2.7
	7	40.4	57.6	2.0
	14	41.4	56.5	2.2
	28	42.4	54.9	2.8
BL(ST)	0	59.5	37.3	3.2
	7	62.1	37.9	0
	16	60.9	39.1	0
	28	61.1	39.2	0

BL—conventional bilberry extract; BL(ST)—solvothermal bilberry extract; DW—dry weight.

**Table 4:** Textural parameter of MCM-41-type supports.

Support	OG/SiO_2_	*S*_BET_ (m^2^/g)	*V*_pore_ (cm^3^/g)	*d*_DFT_ (nm)
MCM-41	-	989	1.59	4.15
MCM-COOH	0.046	628	0.93	4.10
MCM-SH	0.17	933	1.19	2.75

OG—organic group; *d*_DFT_—average pore diameter; *S*_BET_—specific surface area (BET method); *V*_pore_—total pore volume at P/P_0_ = 0.99.

**Table 5:** Extract content of the bilberry-loaded materials.

Sample	Extract (%wt.)	Support (%wt.)	Humidity (%wt.)
BL@MCM-41	39.4	58.3	2.3
BL@MCM-COOH	34.4	64.1	1.5
BL@MCM-SH	20.2	77.9	1.9
BL@Fuc@MCM-SH	21.3	76.0	2.7
BL(ST)@MCM-41	41.6	56.0	2.4

**Table 6:** IC_50_ values of the reference drug (Indomethacin), BL@MCH-SH, and BL against COX-1 and COX-2 and the computed selectivity index (SI).

Sample ID	IC_50_		SI (IC_50_ COX-1/IC_50_ COX-2)
	COX-1	COX-2	
Indomethacin (Reference drug)	0.59 ± 0.1	0.6 ± 0.02	0.98
BL@MCH-SH	1.87 ± 0.06	0.69 ± 0.03	2.71
BL	3.97 ± 0.07	0.97 ± 0.05	4.09

Data are presented as triplicates—mean ± standard deviation (SD). Values in µg sample/mL media.

## Data Availability

All data are presented in the article.

## Supplementary Materials

The following supporting information can be downloaded at https://www.mdpi.com/article/10.3390/antiox13020250/s1. Figure S1: HPLC-PDA analyses for bilberry extracts recorded at 326 nm (1—gallic acid; 2—protocatechuic acid; 3—chlorogenic acid; 4—delphinidin; 5—cyanidin; 6—rutin hydrate; 7—myricetin; 8—*trans*-resveratrol); Figure S2: Thermogravimetric analyses (A) and differential thermal analyses (B) of extract-loaded silica-type supports; Figure S3: Videos captured from in situ microscopy performed during DSC analysis of BL extract (A) and BL@MCM-41 (B); Table S1: Identification of anthocyanin glycosides in BL extracts and their retention time and molecular weight.
