# Supplementary material for: The Antioxidant and Anti-Inflammatory Properties of Wild Bilberry Fruit Extracts Embedded in Mesoporous Silica-Type Supports: A Stability Study
Source: Antioxidants (Basel). 2024 Feb 19;13(2):250. doi: 10.3390/antiox13020250 (PMC10886266; doi:10.3390/antiox13020250)
Supplement: Supplementary file 1 [file antioxidants-13-00250-s001.zip › antioxidants-2858286-supplementary.pdf]

Antioxidant and Anti-inflammatory Properties of Wild Bilberry Fruit Extracts Embedded in Mesoporous Silica-type Supports. A Stability Study

Ana-Maria Brezoiu[1], Mihaela Deaconu[1], Raul-Augustin Mitran[2], Nada K. Sedky[3], Frédéric Schiets[4], Pedro Marote[4], Iulia-Stefania Voicu[1], Cristian Matei[1], Laila Ziko[3] and Daniela Berger[1,*]

[1]National University of Science and Technology Politehnica Bucharest, Faculty of Chemical Engineering and Biotechnologies, 1-7 Gheorghe Polizu Street, 011061 Bucharest, Romania; ana_maria.brezoiu@upb.ro (A.-M.B.), mihaela.deaconu@upb.ro (M.D.), iulia.voicu@stud.upb.ro (I.-S.V.), cristian.matei@upb.ro (C.M.).
[2]"Ilie Murgulescu" Institute of Physical Chemistry, Romanian Academy, 202 Splaiul Independentei, 060021 Bucharest, Romania; raul.mitran@gmail.com.
[3]Department of Biochemistry, School of Life and Medical Sciences, University of Hertfordshire Hosted by Global Academic Foundation, R5 New Garden City, New Administrative Capital, Cairo 11835, Egypt; nadasedky22@gmail.com (N.K.S.), l.adel@gaf.edu.eg (L.Z.)
[4]University Claude Bernard Lyon 1 ISA, UMR 5280 CNRS, 5 rue de la Doua 69100, Villeurbanne, France; pedro.marote@univ-lyon1.fr (P.M.); frederic.schiets@isa-lyon.fr (F.S.).
[*]Authors to whom correspondence should be addressed. Correspondence: daniela.berger@upb.ro;

- Chemicals

Several standard substances were used that were of the high-performance liquid chromatography (HPLC) grade: gallic acid (98%, Alfa Aesar, Ward Hill, MA, USA), protocatechuic acid (>98%, HPLC, TCI, Tokyo, Japan), caftaric acid (Molekula GmbH, Munich, Germany), vanillic acid (>98%, GC, TCI, Tokyo, Japan), syringic acid (>98.5%, Molekula GmbH, Munich, Germany), caffeic acid (98%, HPLC, Sigma, Merck Group, Darmstadt, Germany), trans-*p*-coumaric acid (analytical standard, Sigma-Aldrich Co. Merck Group, Darmstadt, Germany), trans-ferulic acid (>98%, GC) and chicoric acid (>98%) from TCI (Tokyo, Japan), chlorogenic acid (primary reference standard (HWI group, Alpen Aan de Rijn, The Netherlands)), rosmarinic acid (>98%, HPLC, Sigma, Merck Group, Darmstadt, Germany), catechin hydrate (>98%, HPLC, Sigma, Merck Group, Darmstadt, Germany), (−) epicatechin (>98%, HPLC, TCI, Tokyo, Japan), quercetin (>95%, HPLC), rutin hydrate (95%, HPLC), myricetin (>96%, HPLC-grade), kaempferol (>97%, HPLC) from Sigma (Merck Group, Darmstadt, Germany), ellagic acid dihydrate (>98%, HPLC, TCI, Tokyo, Japan), trans-resveratrol (certified reference material, Sigma-Aldrich Co. Merck Group, Darmstadt, Germany); anthocyanidins: cyanidin chloride (>95%, HPLC, Sigma, Merck Group, Darmstadt, Germany), malvidin chloride (>95%, HPLC, Sigma-Aldrich Co. Merck Group, Darmstadt, Germany), pelargonidin chloride (Aldrich Chemical Co Inc., Milwaukee, WI, USA), and delphinidin chloride (analytical standard, Sigma-Aldrich Co. Merck Group, Darmstadt, Germany); and solvents, including ethanol and acetonitrile (ACN), were purchased from Riedel-de Haën, Honeywell Riedel-de Haën, Seelzer, Germany, and formic acid (Merck Group, Darmstadt, Germany) was used without additional purification. For all solutions and experiments, ultrapure water (Millipore Direct- Q3 ultraviolet (UV) water purification system with Biopack UF cartridge) was used.

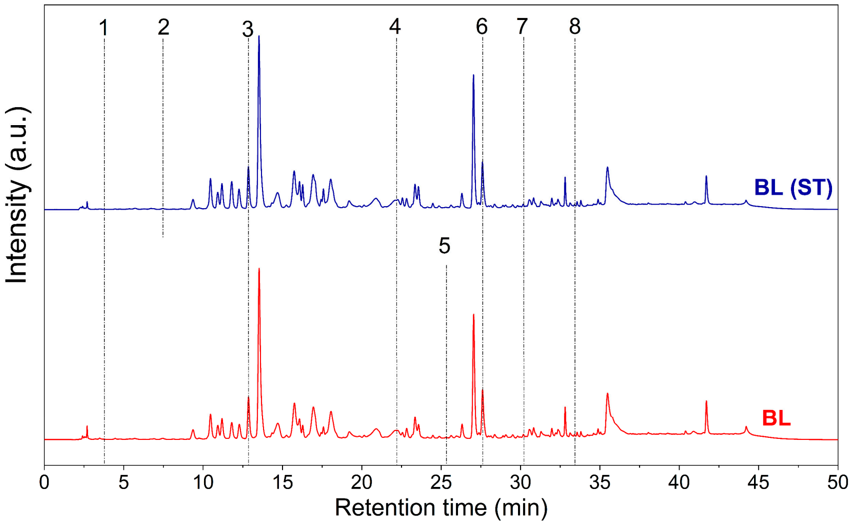

Figure S1. HPLC-PDA analyses for bilberry extracts recorded at 326 nm (1—gallic acid; 2—protocatechuic acid; 3—chlorogenic acid; 4—delphinidin; 5—cyanidin; 6—rutin hydrate; 7—myricetin; 8—trans-resveratrol).

Extract-loaded materials were evaluated by thermogravimetric analyses and radical scavenger activity on solid samples. Based on thermogravimetric analyses (Figure S2), the content of polyphenolic compounds was determined by deducting the weight loss of both adsorbed water and functional groups grafted on silica. The extract-loaded materials have a content of phytocompounds in the range of 20.2-41.6%wt.

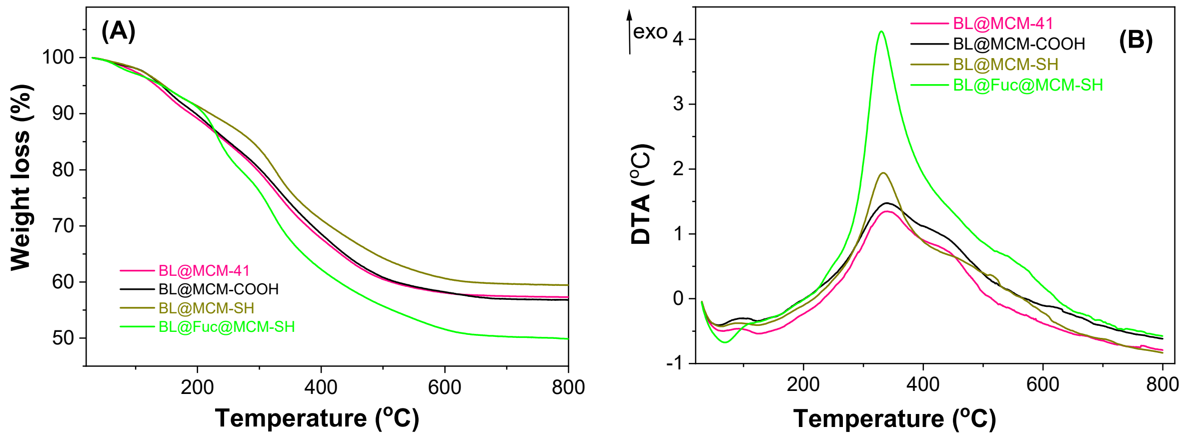

Figure S2. Thermogravimetric analyses (**A**) and differential thermal analyses (**B**) of extract-loaded silica-type supports.

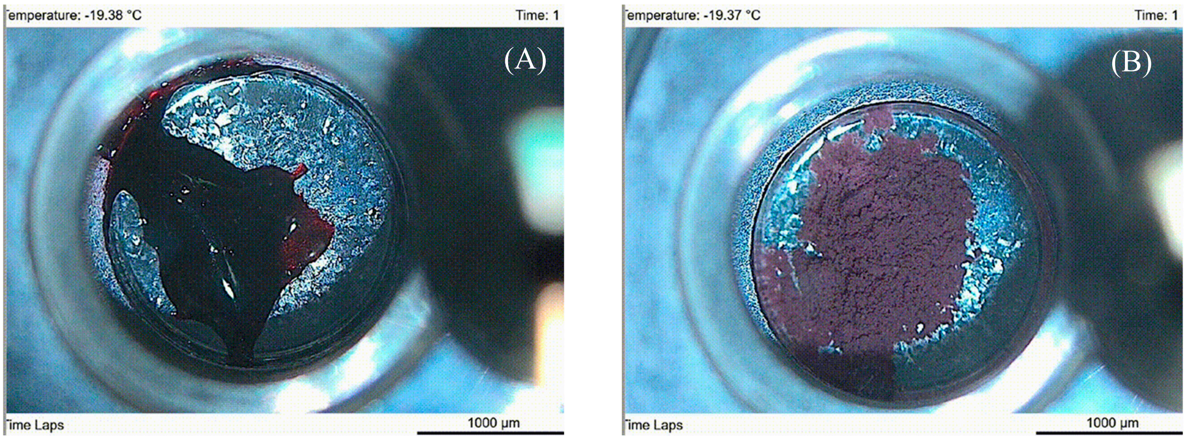

Figure S3. Videos captured from in situ microscopy performed during DSC analysis of BL extract (A) and BL@MCM-41 (B)

Table S1. Identification of anthocyanin glycosides in BL extracts and their retention time and molecular weight.

Tentative identification	RT	MW (g/mol)	MS (m/z)
Delphinidin-3-*O*-galactoside	3.14	465	464[M-H]$^-$
Delphinidin-3-*O*-glucoside	3.56	465	464[M-H]$^-$
Cyanidin-3-*O*-galactoside	4.27	449	448[M-H]$^-$
Delphinidin-3-*O*-arabinoside	4.34	435	434[M-H]$^-$
Cyanide-3-*O*-glucoside	5.08	449	448[M-H]$^-$
Cyanid-3-*O*-arabinoside	6.22	419	418[M-H]$^-$
Petunidin-3-*O*-glucoside	6.91	479	478[M-H]$^-$
Malvidin-3-*O*-galactoside	12.60	493	492[M-H]$^-$

RT—retention time
